# Immunization against a Saccharide Epitope Accelerates Clearance of Experimental Gonococcal Infection

**DOI:** 10.1371/journal.ppat.1003559

**Published:** 2013-08-29

**Authors:** Sunita Gulati, Bo Zheng, George W. Reed, Xiaohong Su, Andrew D. Cox, Frank St. Michael, Jacek Stupak, Lisa A. Lewis, Sanjay Ram, Peter A. Rice

**Affiliations:** 1 Department of Medicine, Division of Infectious Diseases and Immunology, University of Massachusetts Medical School, Worcester, Massachusetts, United States of America; 2 Department of Medicine, Division of Preventive and Behavioral Medicine, University of Massachusetts Medical School, Worcester, Massachusetts, United States of America; 3 Institute of Dermatology, Chinese Academy of Medical Sciences, Nanjing, People's Republic of China; 4 Vaccine Program, Human Health Therapeutics Portfolio, National Research Council Canada, Ottawa, Ontario, Canada; Faculté de Médecine Paris Descartes, site Necker, France

## Abstract

The emergence of ceftriaxone-resistant strains of *Neisseria gonorrhoeae* may herald an era of untreatable gonorrhea. Vaccines against this infection are urgently needed. The 2C7 epitope is a conserved oligosaccharide (OS) structure, a part of lipooligosaccharide (LOS) on *N gonorrhoeae*. The epitope is expressed by 94% of gonococci that reside in the human genital tract (*in vivo*) and by 95% of first passaged isolates. Absence of the 2C7 epitope shortens the time of gonococcal carriage in a mouse model of genital infection. To circumvent the limitations of saccharide immunogens in producing long lived immune responses, previously we developed a peptide mimic (called PEP1) as an immunologic surrogate of the 2C7-OS epitope and reconfigured it into a multi-antigenic peptide, (MAP1). To test vaccine efficacy of MAP1, female BALB/c mice were passively immunized with a complement-dependent bactericidal monoclonal antibody specific for the 2C7 epitope or were actively immunized with MAP1. Mice immunized with MAP1 developed a T_H_1-biased anti-LOS IgG antibody response that was also bactericidal. Length of carriage was shortened in immune mice; clearance occurred in 4 days in mice passively administered 2C7 antibody vs. 6 days in mice administered control IgG3λ mAb in one experiment (p = 0.03) and 6 vs. 9 days in a replicate experiment (p = 0.008). Mice vaccinated with MAP1 cleared infection in 5 days vs. 9 days in mice immunized with control peptide (p = 0.0001 and p = 0.0002, respectively in two replicate experiments). Bacterial burden was lower over the course of infection in passively immunized vs. control mice in both experiments (p = 0.008 and p = 0.0005); burdens were also lower in MAP1 immunized mice vs. controls (p<0.0001) and were inversely related to vaccine antibodies induced in the vagina (p = 0.043). The OS epitope defined by mAb 2C7 may represent an effective vaccine target against gonorrhea, which is rapidly becoming incurable with currently available antibiotics.

## Author Summary

*Neisseria gonorrhoeae* cause a common sexually transmitted infection (ca. 106 million cases per year, worldwide). Gonococcal organisms have become resistant to almost all previously effective antibiotics, creating an urgent need for preventive vaccines. Extreme variability of the gonococcal surface has precluded identification of common components that might be present on a wide variety of isolates/strains; these might serve as effective vaccine targets. We identified a common sugar structure, present on 95% of gonococcal organisms; this structure elicits a large and specific antibody response in women after natural gonococcal infection. Because peptides are more effective than sugars when used as vaccines, we identified peptide “look-alikes” that mimicked the sugar; first by using a monoclonal antibody that recognized the sugar, then by screening trillions of small peptides to identify those also recognized by the antibody. Mice that were vaccinated with one of these peptides cleared infection more quickly than animals vaccinated with an irrelevant peptide. We also found that administration of antibody from immune to normal, unimmunized, mice, prior to infection, subsequently hastened clearance of infection, indicating that the antibody administered was the protective agent. This study represents an important step in developing a vaccine to protect humans from infection caused by a wide variety of gonococcal strains.

## Introduction

*Neisseria gonorrhoeae* infection is the second most common bacterial sexually transmitted infection (STI); the worldwide incidence is 106 million cases per year [1]. Gonococci cause a broad spectrum of diseases [2]; HIV co-infection in men enhances risk of HIV transmission to female sex-partners [3]. Recent, widespread emergence of resistance to currently used antimicrobials [4] and the potential for spread of resistant gonococci threaten to herald an era of untreatable disease, worldwide. Uniform vaccination of persons at greatest risk would be an effective deterrent.

Development of safe effective vaccines against gonococcal infection is challenging because the correlates of immune protection are not fully known [5]. Furthermore, gonococcal surface molecules that may be appropriate targets often are antigenically variable. Unfortunately, adaptive immune responses that target highly conserved gonococcal antigens fail to elicit protection [6].

*N. gonorrhoeae* lipooligosaccharide (LOS) is an important component of the gonococcal outer membrane [7]. Antibodies directed against LOS engage complement to kill *N. gonorrhoeae* directly [8] and also promote opsonophagocytosis [9]. LOS antibodies may also contribute to protection against re-infection with the homologous strain in experimental infection of human male volunteers [10].

**Figure 1:**
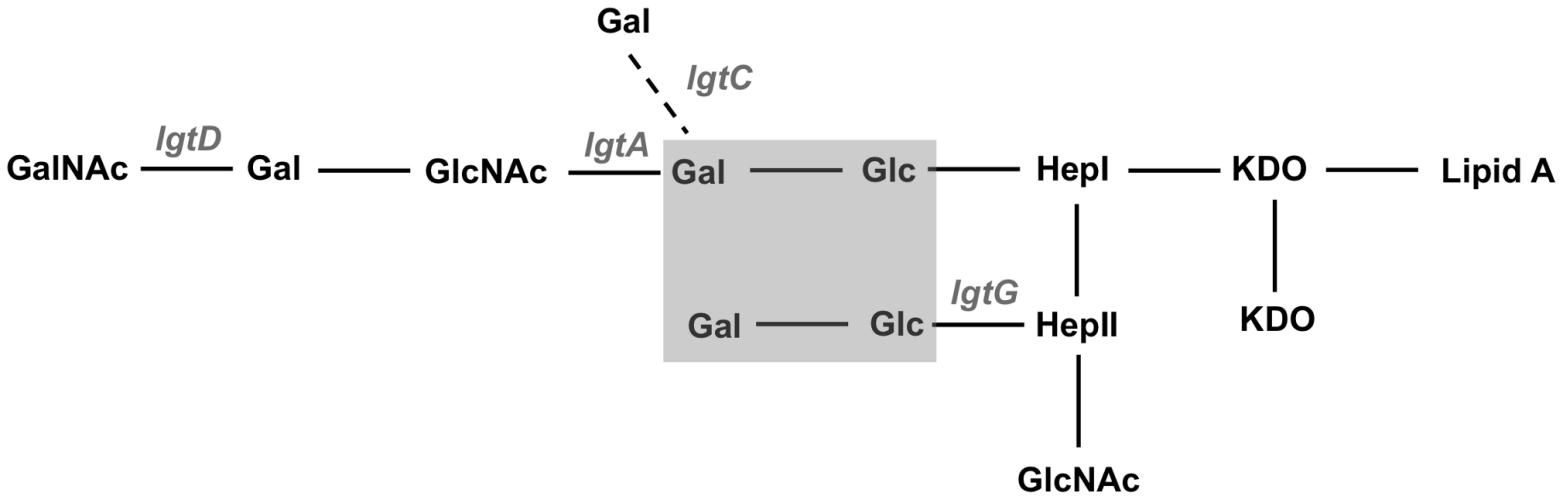
Simplified schematic of the oligosaccharide (OS) structure of gonococcal LOS. Lipooligosaccharide glycosyl transferase *(lgt)* genes that are involved in LOS biosynthesis and are subject to phase variation are indicated in grey italic font. Each transferase catalyzes the substitution of the next more distal hexose onto the LOS backbone. The grey shaded box represents the minimum OS structure expressed by naturally occurring gonococcal strains that is required for mAb 2C7 binding.

Despite antigenic heterogeneity of LOS, we have identified a common oligosaccharide structure within gonococcal LOS that is recognized by a murine monoclonal antibody (mAb), called 2C7 [9], [11]. This structure (Figure 1) requires the substitution of lactose onto HepII and at a minimum, substitution of lactose on HepI [12]. The 2C7 epitope was identified directly in the genital secretions of 94% of 68 culture-positive subjects and on 95% of 101 strains of *N. gonorrhoeae* isolated from infected subjects [9]. Human antibodies against the 2C7 epitope also mediate complement-dependent bacterial killing and opsonophagocytosis. Compared to purified LOS, the 2C7 epitope selectively elicited a greater antibody response after gonococcal endometritis and disseminated infection [9]. Male volunteers immunized with a gonococcal outer membrane vaccine that contained LOS harboring the 2C7 epitope developed a 10-fold excess of 2C7 antibody compared to a rise in antibody against whole LOS [9], thereby confirming superior immunogenicity of the 2C7 epitope in a human vaccine trial.

Carbohydrates are poor immunogens and induce T cell-independent immune responses that may not lead to full affinity maturation and are poor immunogens [13]. A promising approach in vaccine design uses peptides that are the structural and/or functional mimics of carbohydrate antigens [14], [15], [16]. Peptide mimics elicit cross-reactive immune responses to the nominal carbohydrates [17]; when used for immunization they can elicit an immune response against carbohydrate antigens and lead to effective immunity [17].

Previously, we selected a peptide mimic of the 2C7 epitope by screening a random peptide library with mAb 2C7 [11]. We reconfigured the peptide into a multi-antigenic form, called MAP1 [11]. Immunization of mice elicited cross-reactive anti-LOS antibodies that possessed dose-responsive direct complement-dependent bactericidal activity against gonococci [11]. Here, we further characterized the MAP1 induced antibody responses in mice and determined the efficacy of both passive immunization with mAb 2C7 and active vaccination with MAP1 in attenuating infection in mice following experimental vaginal challenge with *N. gonorrhoeae.*

## Results

### Characterization of LOS structure of gonococcal strains: FA1090wt and FA1090*lgtG^−^*

Figure 1 illustrates gonococcal LOS structure identifying the innermost oligosaccharide structures that bind mAb 2C7 (shaded gray) and the phase-variable LOS glycosyl transferases (*lgt*) biosynthetic genes. Loss of lactose (Gal-Glc) substitution on HepII in the FA1090*lgtG^−^* (*lgtG^−^*) mutant was confirmed by compositional analysis and by loss of reactivity with mAb 2C7 in western blot and whole-cell ELISA (data not shown). The “wild-type” (wt) and *lgtG^−^* mutant expressed similar HepI LOS substituted glycan extensions, revealed by whole-cell ELISA and western blot using 3 mAbs that recognize distinct HepI glycan extensions (Figure 1): 3F11 (lacto-*N*-neotetraose), L8 (lactose) and L1 (Gal→Gal→Glc, or the P^K^-like structure [18], [19], [20], [21] (data not shown). Furthermore, DNA sequencing indicated that there was no variation in the homopolymeric regions of *lgtA*, *lgtC* and *lgtD* (Figure 1) between the wt and *lgtG^−^* mutant suggesting that expression of HepI glycan extensions in the wt and mutant were similar (data not shown).

### Loss of *lgtG* expression attenuates *N. gonorrhoeae* FA1090 infection

The 2C7 epitope is expressed on most gonococci that infect humans [9] and we hypothesized that the phase-variable *lgtG* gene remains in-frame (on) because it provides the organism a survival advantage in its natural (human) host. Growth and viability of FA1090wt and FA1090*lgtG* ^−^ were similar *in vitro* as revealed by similar growth curves (doubling time and final optical density) when the strains were grown separately or together in co-culture (data not shown). To establish the role of *lgtG in vivo*, mice were inoculated with a mixture of strains FA1090wt (1.5×10^5^ colony forming units [CFU]) and FA1090*lgtG^−^* (1.6×10^5^ CFU). Five of 5 inoculated mice became infected. Kaplan Meier analysis of time to median clearance of FA1090*lgtG^−^* was 4 days; only one mouse cleared FA1090wt infection by 10 days, the length of the experiment (*P* = 0.04), (Figure S1A). A lower longitudinal trend in mean log_10_ CFU colonization over time was measured for the *lgtG^−^* mutant (Figure S1B) (*P*<0.001). Cumulative CFU expressed as area under the curve (AUC) also showed a lower bacterial burden for the *lgtG^−^* mutant strain (*P* = 0.04), (Figure S1C). Based on the *in vivo* co-culture results, we hypothesized that although selective (antibody) pressure targeting the 2C7 epitope may serve to select for phase variation events that yield a 2C7 epitope negative gonococcal infection, the reduced fitness associated with loss of the 2C7 epitope would not favor continued infection.

### mAb 2C7 accelerates clearance of HepII lactose bearing gonococci

**Figure 2:**
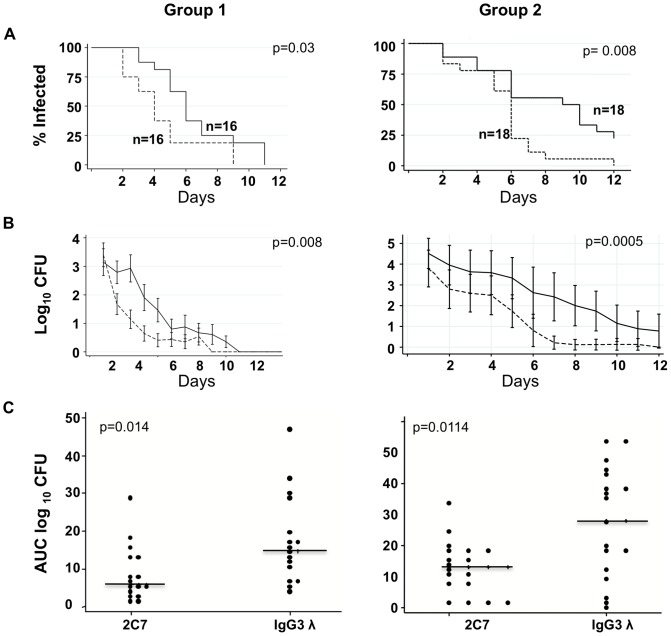
Survival of FA1090wt inoculated into mice passively immunized with mAb 2C7. mAb 2C7 (dashed lines in ***A*** **.** and ***B.***) vs. control IgG3λ mAb (solid lines) treated animals followed by challenge with FA1090wt. Two separate experiments were performed; Group 1 and Group 2 (n = number of infected mice in each group) as indicated. ***A*** *.* Kaplan Meier analysis of time to clearance of FA1090wt showing differences in clearance of mAb 2C7 vs. control IgG3λ mAb treated animals. ***B*** *.* Colonization of FA1090wt (Log_10_ CFU) measured daily in mAb 2C7 vs. control IgG3λ mAb treated animals. **C**. Bacterial burdens consolidated over time (Area Under the Curve [log _10_ CFU] analysis) of mAb 2C7 vs. control IgG3λ mAb treated animals.

We examined whether passive administration of mAb 2C7 also shortens time of FA1090wt infection in mice. Mice were administered mAb 2C7 or control IgG3λ mAb intraperitoneally (ip) daily for 3 days prior to and including the day of challenge. In Group 1 (Figure 2), using a vaginal inoculum of 5×10^5^ CFU, 16 of 20 (80%) mice administered mAb 2C7 and 16 of 20 mice administered control IgG3λ mAb became infected. In Group 2 (Figure 2), using a 3.6×10^5^ CFU inoculum; 18 of 20 (90%) mice administered mAb 2C7 and 18 of 20 mice given control IgG3λ mAb became infected.

Kaplan Meier analysis showed that mAb 2C7-treated animals cleared gonococci faster than IgG3**λ** mAb-treated control animals in both experiments (Experiment 1: median time to clearance; 4 days in mAb 2C7 administered vs. 6 days in the IgG3**λ** mAb administered group, *P* = 0.03 [Figure 2A, left panel]; Experiment 2: median time to clearance; 6 days in mAb 2C7 administered vs. 9 days in the IgG3**λ** mAb administered group, *P* = 0.008 [Figure 2A, right panel]). A diminished longitudinal trend in mean log_10_ CFU colonization over time was measured in mAb 2C7 administered mice (*P* = 0.008 and *P* = 0.0005 in Experiments 1 and 2, respectively [Figure 2B]). Cumulative CFU expressed as area under the curve (AUC) was also lower in mAb 2C7 administered mice (*P* = 0.014 and *P* = 0.0114 in Experiments 1 and 2, respectively [Figure 2C]). All gonococcal colonies isolated from the 2 mice that remained infected at 7 days in Experiment 2 after inoculation (75 and 40 colonies per mouse) were detected by mAb 2C7 in colony blots further emphasizing that the 2C7 structure is important for prolonged survival of FA1090 in the mouse model.

Clearance of the FA1090*lgtG* ^−^ mutant occurred similarly in single infection in the presence of either mAb 2C7 or control IgG3**λ** mAb (Figure S2). There were no differences between the two groups in median time to clearance (5 days in both groups; *P* = 0.86 by Kaplan Meier analysis [Figure S2A]), mixed model analysis (*P* = 0.95) (Figure S2B) and AUC analysis (*P* = 0.56) (Figure S2C).

### Antibody elicited following MAP immunization

**Figure 3:**
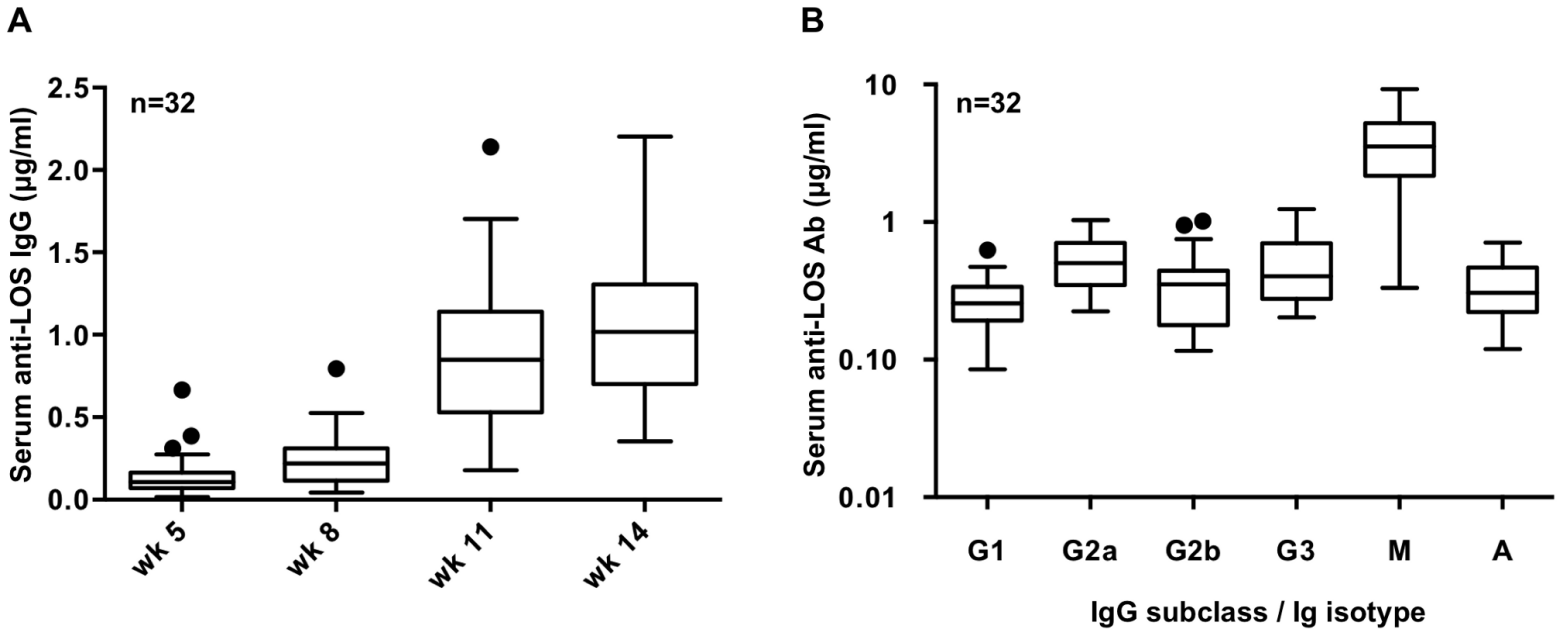
Anti-LOS IgG antibody responses induced by MAP1-MPL immunization. 32 BALB/c mice were immunized intraperitoneally (ip) with MAP1 emulsified with MPL and boosted four times at 3-week intervals. ***A.*** Total anti-LOS IgG antibody levels at wk 5, 8,11 and 14 following immunization. ***B.*** Post-immunization (wk 14) anti-LOS IgG subclass, IgM and IgA anti-LOS antibody levels. Results are represented as box plots; the spots represent outliers beyond the 1.5 IQR (intraquartile range). IgG subclass responses indicate a T_H_1-biased response (see text, [22]).

Sera from mice actively immunized with MAP1 or MAP-control (a peptide that does not bind 2C7), each administered with a Monophosphoryl Lipid A (MPL) containing adjuvant, were evaluated for total IgG antibody elicited against LOS of FA1090wt. Two weeks following the third booster of MAP1 (given in week 14), the median serum anti-LOS IgG antibody level had risen to 1.018 µg/ml (range 0.353 to 2.204 µg/ml) (Figure 3A). A mixed model analysis of mean anti-LOS antibody levels over time showed significant increases between weeks designated for antibody testing (*P*<0.001) (Figures 3A and S3). MAP-control immunization did not yield anti-LOS IgG responses (<0.001 µg/ml) but showed IgG antibody responses against MAP-control itself (data not shown). MAP1 immune sera did not contain detectable IgG antibody (<0.001 µg/ml) against LOS prepared from FA1090*lgtG* ^−^, confirming that the LOS antibody response following MAP1 immunization was 2C7 specific (data not shown). Samples obtained from vaginal swabs showed median anti-LOS IgG levels of 0.008 µg/ml (range 0.005–0.015 µg/ml) from MAP1 immunized mice at 14 weeks (data not shown).

Serum anti-LOS IgG subclass levels, anti-LOS IgM and IgA levels in the 32 mice immunized with MAP1 were quantitated at week 14 after immunization (Figure 3B). As expected, MAP-control immunized mice did not yield detectable anti-LOS antibodies. Subclass analysis of the anti-LOS response suggested a T_H_1-biased response (T_H_1:T_H_2 index response as defined by the ratio [(IgG2a+IgG3)×0.5]/IgG1 was 1.75; ratios >1 and <1 indicate T_H_1 and T_H_2 responses, respectively [22]).

### Immune anti-LOS IgG mediates anti-gonococcal bactericidal activity

 Immunization of the 32 mice with MAP1-MPL elicited a 3.8-fold higher IgM antibody response against LOS compared to IgG (Figure 3B). Using 17% added human complement, we measured the relative contributions of anti-LOS IgG and anti-LOS IgM to serum bactericidal activity against FA1090wt in serum from 6 separate mice that had been immunized with MAP1-MPL but not challenged with *N. gonorrhoeae*. Non-challenged animals were immunized identically to their challenged counterparts (same lot, time of immunization and schedule); the mean ± SE anti-LOS IgG, IgG subclasses, IgA and IgM levels in the sera of these 6 mice at week 14 were similar to the corresponding Ig levels in mice used for challenge (Table S1). Likewise, the mean differences in Ig levels between the two groups were similar (Table S2). Immune sera from the 6 mice also lacked IgG antibody against FA1090*lgtG* ^−^ LOS.

**Figure 4:**
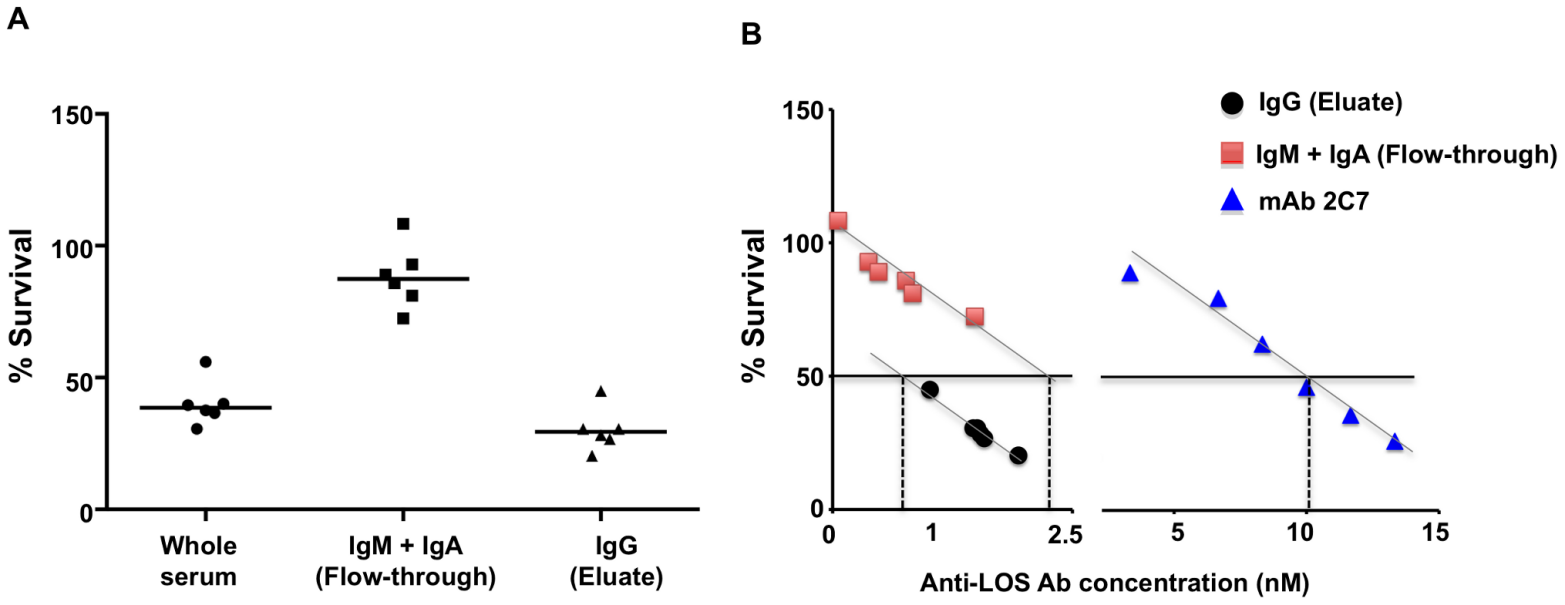
Bactericidal activity of serum fractions directed against FA1090wt from 6 mice immunized with MAP1-MPL. On average, the serum fractions eluted from Protein A/G plus agarose maintained 96% of the anti-LOS IgG concentrations of whole sera. Flow-through fractions maintained 92% and 81% of IgM and IgA anti-LOS concentrations, respectively, as compared to whole sera and contained no IgG (Supplemental Table S3). ***A*** *.* Bactericidal activity of immune Whole serum, IgG-depleted immune serum (labeled IgM + IgA (Flow through)) and immune IgG fraction (labeled IgG (Eluate)) from 6 mice immunized with MAP1-MPL. Results are expressed as % survival of FA1090wt CFUs surviving incubation for 30 min. in a reaction mixture containing 20% serum (each of the 6 immune sera were tested separately) or an amount of each of the corresponding chromatographic fractions that contained the equivalent of the amount of IgG or IgM in 20% of the parent serum. Complement (17% (v/v)) was added separately. Results are expressed as survival at 30 min. compared to survival at 0 min. ***B*** *.* Immune IgG (Eluate) anti-LOS was more bactericidal than immune IgM + IgA (Flow-through) anti-LOS and mAb 2C7, on a nanomolar basis. The percent survival of FA1090wt in the presence of serum fractions from of the each of the 6 mice immunized with MAP1-MPL: (1) immune IgG (Eluate, black circles); (2) IgM + IgA ((Flow-through), red squares) and separately, (3) serial dilutions of mAb 2C7 (blue triangles). Results of each chromatographic fraction or serial dilution of 2C7 mAb were plotted as a function of the anti-LOS IgG, IgM or 2C7 (also IgG) concentrations (nM), respectively. Results are expressed as % survival of FA1090wt CFUs in the presence of immune serum fractions or mAb 2C7 with added complement (17%) at 30 min compared to survival at 0 min. The dashed black vertical lines show the x-intercepts (abscissae) that indicate the concentration of the Anti-LOS antibody concentration (nM) that yielded 50% survival (shown by the solid horizontal black line) of FA1090.

Each of the 6 sera was passed over protein A/G agarose; the flow-through contained IgM and IgA and was devoid of IgG. IgG eluted from the protein A/G column contained IgM at 10%–20% and IgA at 2–15% of serum levels (Table S3). IgG fractions (Figure 4A; labeled IgG (Eluate)) were more bactericidal (29% median survival) than corresponding sera devoid of IgG (Figure 4A; labeled IgM + IgA (Flow-through); 87% median survival), (*P* = 0.0001). Percent survival of gonococci in each of the serum fractions was expressed as a function of the nanomolar concentrations of anti-LOS IgG in the IgG fraction (Eluate) or anti–LOS IgM present in the IgM + IgA fraction (Flow-through; Figure 4B); IgA is not bactericidal. On a nanomolar basis, the anti-LOS IgG fraction (Figure 4B; labeled IgG (Eluate)) was ∼2.9-fold more bactericidal than the anti-LOS IgM fraction (Figure 4B; labeled IgM + IgA (Flow-through)). Both anti- LOS IgG and IgM + IgA fractions showed a significant inverse correlation with bacterial survival (*R^2^* = 0.974 for IgG, *P* = 0.0003 and *R^2^* = 0.877 for IgM, *P* = 0.006).

Having shown that the majority of the killing activity in each of the 6 immune sera was mediated by anti-LOS IgG antibody, we next compared killing by polyclonal immune IgG antibody with killing by mAb 2C7. In the presence of human complement, mAb 2C7 also killed FA1090wt in a concentration-dependent manner (Figure 4B; *R^2^* = 0.9661, *P* = 0.0004,). Using 50% survival in the bactericidal assay as a measure to discriminate bactericidal activity in the different fractions (Figure 4B), on a nanomolar basis, the polyclonal IgG fraction in sera from mice immunized with MAP1-MPL was ∼10–12.5-fold more bactericidal against FA1090wt than mAb 2C7 (Figure 4B).

### Gonococcal infection is attenuated in mice immunized with MAP1

**Figure 5:**
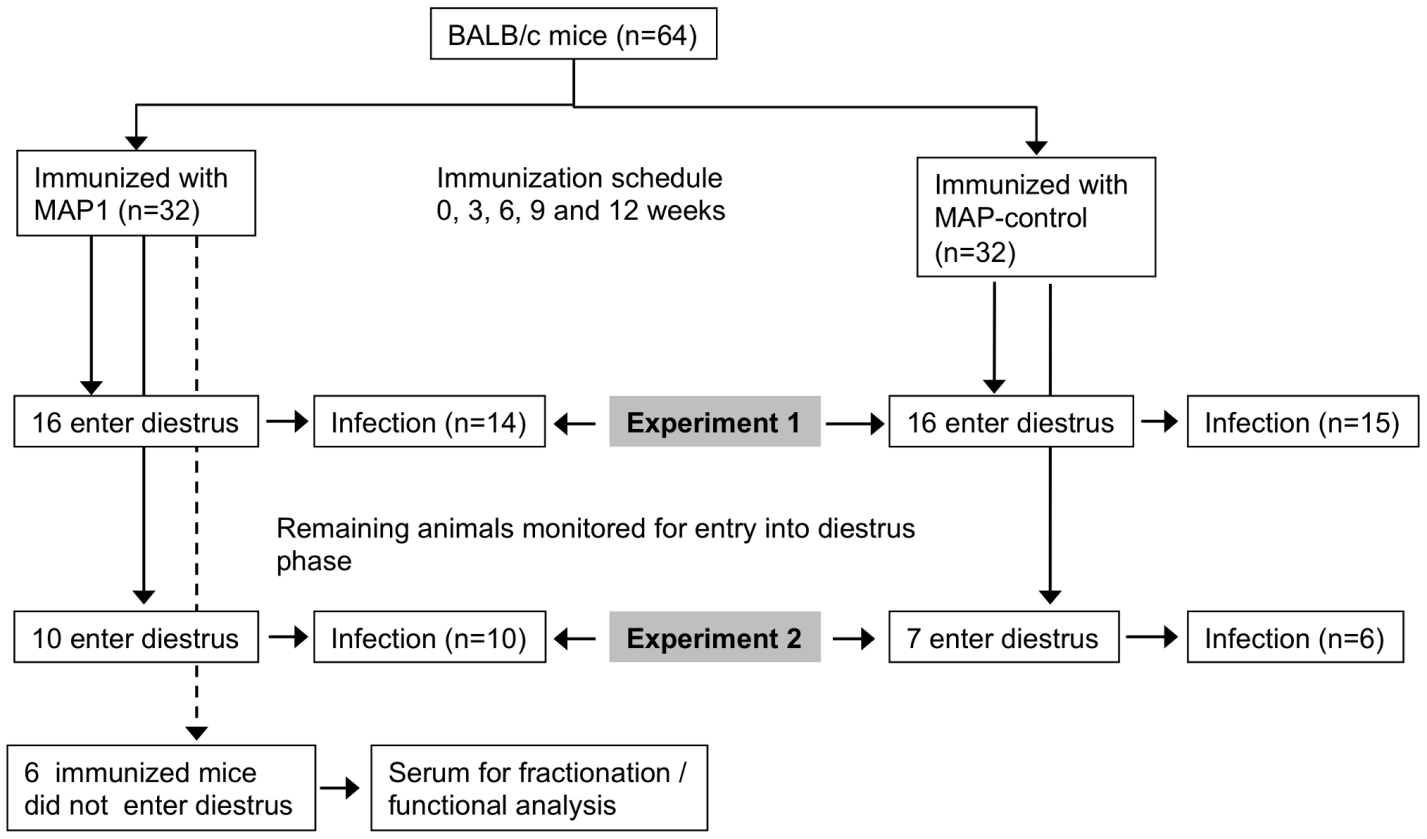
Experimental design of active immunization with MAP1: Schematic representation. Mice entering the diestrus phase of the estrous cycle were started on treatment (that day) with 0.5 mg. of water soluble 17β-estradiol (Sigma) given subcutaneously on each of three days; −2, 0 and +2 days (before, the day of and after inoculation) to prolong the estrus phase of the cycle and promote susceptibility to *N. gonorrhoeae* infection. Antibiotics ineffective against *N. gonorrhoeae* were also used to reduce competitive microflora.

**Figure 6:**
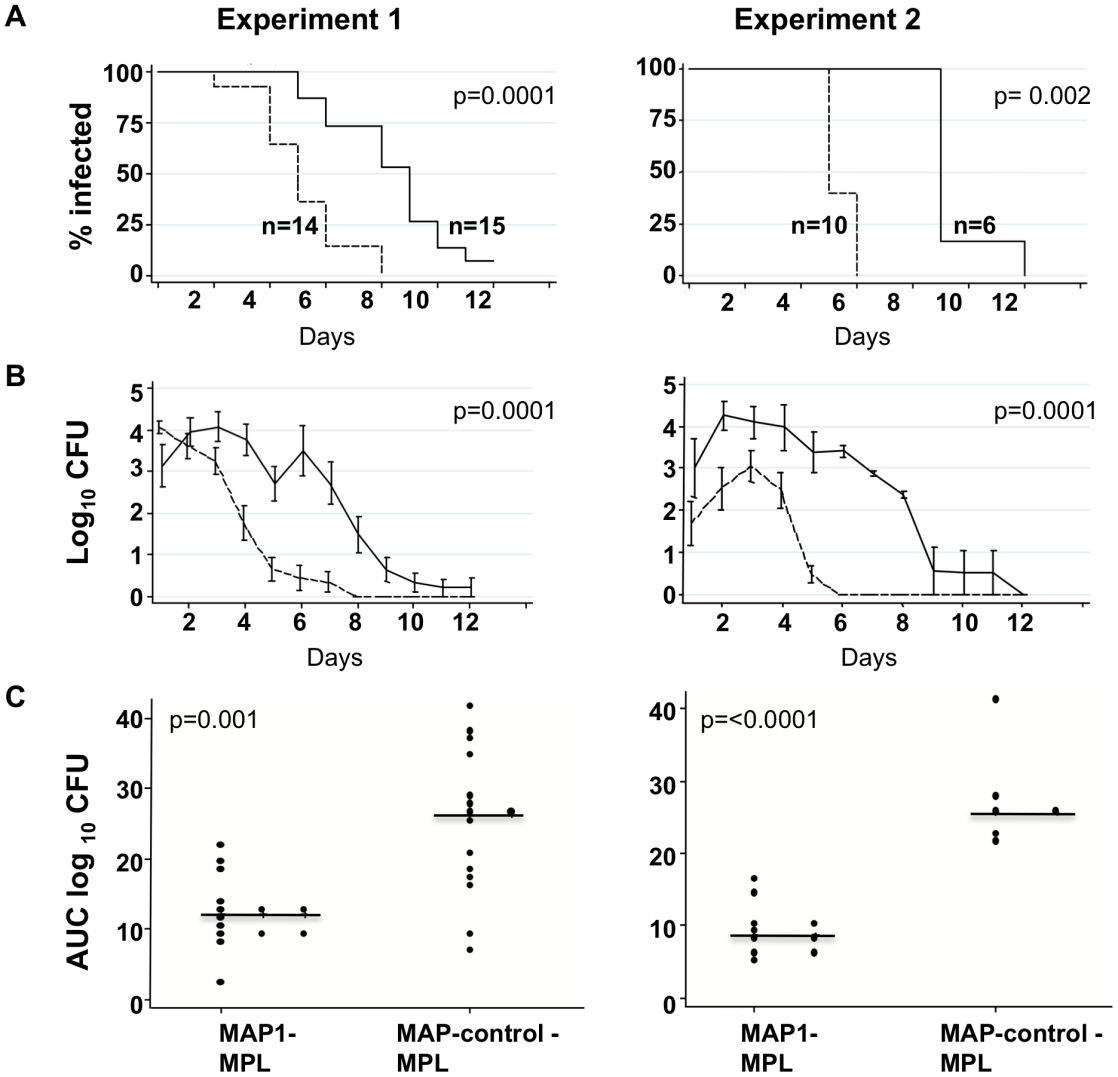
Survival of FA1090wt inoculated into mice actively immunized with MAP1-MPL. Group 1: MAP1-MPL immunized mice (16 immunized, 14 infected) and MAP-control-MPL immunized mice (16 immunized, 15 infected) were challenged with FA1090wt (5.4×10^5^ CFU). Group 2: MAP1 immunized mice (10 immunized, 10 infected) and MAP-control immunized mice (7 immunized, 6 infected were challenged with FA1090wt (4×10^5^ CFU). ***A.*** Kaplan-Meier curves indicating time to clearance of challenged *N. gonorrhoeae* FA1090wt. ***B.*** Mean log_10_ (CFU) isolation trends over time. Isolation of *N. gonorrhoeae* from MAP1 and MAP-control immunized mice are represented by dashed and solid lines, respectively. ***C.*** Mean area under the curve (AUC log_10_ CFU) vs. time computed for each mouse to consolidate cumulative infection; AUC log_10_ CFU was compared between groups.

In Groups 1 and 2 (Experimental Design shown in Figure 5), MAP1 and MAP-control immunized mice (n = 16 in each group in Group 1 and n = 10 and n = 7 respectively, in Group 2) were selected/pre-treated as described in Materials and Methods and then challenged intravaginally with FA1090wt (5.4×10^5^ CFU in Group 1 and 4×10^5^ CFU in Group 2). Fourteen of 16 mice from the MAP1 group in Group 1, 10/10 mice in Group 2 and 15/16 mice from the MAP-control group in Group 1 and 6/7 mice in Group 2 became infected at the outset (defined by a positive intravaginal culture for *N. gonorrhoeae* on Day 1 and/or 2). Time to clearance of FA1090wt by Kaplan Meier was decreased in MAP1 vs. MAP-control immunized mice (median time of clearance, 5 vs. 9 days) in each experiment (*P* = 0.0001 and *P* = 0.002 in Groups 1 and 2, respectively) (Figure 6A). Mixed model analysis indicated significant differences in colonization trends of FA1090wt over time between the two groups comparing MAP1 vs. MAP-control immunized mice in both Groups 1 (*P* = 0.0001) and 2 (*P*<0.0001) (Figure 6B). A significant difference in the Mean Areas Under the Curve (MAUCs) (log_10_ CFU vs. time) between groups inoculated with FA1090wt was seen in each of the challenge experiments: *P* = 0.001 in Experiment 1 and *P*<0.0001 in Experiment 2 (Figure 6C).

In the twenty four animals that became infected at the outset, in Groups 1 and 2 combined, vaginal antibody concentrations varied inversely with the burden of infection (Area Under the Curve); p = 0.043.

## Discussion

*N. gonorrhoeae* possess numerous mechanisms to subvert innate immune mechanisms [23], [24] and adaptive immune responses following natural infection (reviewed in [25]). Lack of serum bactericidal activity in infected persons [26] may result from the presence of “blocking” (subversive) antibodies against Rmp, a conserved antigenic structure in the gonococcal outer membrane [27]. In addition, engagement of carcinoembryonic antigen cellular adhesion molecule 1 (CEACAM1) on lymphocytes may suppress CD4 T cell responses [28] that can augment or suppress the development of protective immunity. In a murine infection model [29], gonococci incite a T_H_17 response that promotes an influx of PMNs that may serve as sanctuary for live gonococci that gain entry via opsonic mechanisms [30]. There are a numerous host factors in humans that influence gonococcal infection and may restrict infection in the mouse model of infection [31] A major restriction that is relevant to the IgG antibody protection model includes complement regulators, such as factor H and C4b-binding protein (C4BP) that down regulate IgG mediated complement activation in humans but do not bind to *N. gonorrhoeae* in the mouse and therefore do not exhibit regulator function [32], [33].

*In vitro* correlates of protection of gonococcal vaccines have not been defined. Antibodies against serovar defining porin (Por) types may be associated with a decreased risk of re-infection with *N. gonorrhoeae* [34], although infected subjects were not fully protected from re-infection with the same Por type [35]. Antibodies against gonococcal opacity associated (Opa) proteins, which are phase-variable and lack serovar specificity [36], [37], were associated with diminished rates of gonococcal salpingitis [37]. In contrast, antibodies against Rmp, which is conserved, were associated with enhanced likelihood of gonococcal infection in commercial sex workers in Kenya [27]. It is noteworthy that anti-Rmp antibodies block killing of gonococci by bactericidal antibodies [38].

It is no surprise that a safe and effective vaccine against *N. gonorrhoeae* has remained elusive. Early efforts using killed bacteria did not confer protection in humans [39]. Subsequently, pilin (Pil) derived from a single strain was effective in protecting male human volunteers from experimental urethral infection, but only against the homologous strain [40]. This Pil vaccine failed to protect U.S. military personnel stationed in Korea because of the high degree of antigenic variation in Pil across strains [41]. An outer membrane vaccine enriched with porin elicited antibody responses against Rmp [42] and 2C7 [9] that were disproportionately increased compared to porin antibody. The vaccine failed to protect against experimental challenge with the strain used to prepare the vaccine, principally, it was thought, because of the excessive/subversive Rmp response [42] Nevertheless, immunogenicity of the 2C7 epitope in this vaccine trial and also in natural infection [9] spurred our efforts to develop a peptide mimic of 2C7 as a vaccine candidate.

The 2C7 mimic displayed as a multi-antigen peptide elicited a ≥4-fold increase in anti-LOS titers in 87% of immunized BALB/c mice [11]. In the current study, we showed that either passive immunization with mAb 2C7 that targets the epitope or immunization with the 2C7 mimic, MAP1, shortens the course of gonococcal infection in the mouse model of genital infection. Vaginal levels of vaccine induced 2C7 immune antibodies correlated significantly with reduction in bacterial burden. These results strongly support an antibody–mediated effect that was dependent on the presence of local IgG antibody in mouse vaginas.

Greater specific bactericidal activity of IgG antibody in immune sera compared to mAb 2C7 (Figure 4B) may have been related to a predominant complement-activating IgG subclass (IgG2a) response (Table S3) resulting from T_H_1-biased immune stimulation elicited by MAP-1 used in combination with MPL, a known T_H_1 adjuvant [43]. Simultaneous elicitation of IgG3 LOS antibody indirectly confirmed that the peptide immunogen indeed acted as a carbohydrate mimotope [13].

Immunization with MAP-1 MPL also elicited an anti-LOS IgM response. Specific bactericidal activity of anti-LOS IgM was ∼3-fold less than anti-LOS IgG but accounted for one-third of bactericidal activity in vaccinated mice. Pentavalent IgM is the most potent complement activating class of antibody. We speculate that the lesser role of anti-LOS IgM bactericidal function, compared to anti-LOS IgG, may have resulted from the recruitment of complement fixing IgG2b LOS antibodies (in addition to the predominant IgG2a) by MAP1-MPL. Diminished bactericidal function of IgM anti-LOS antibody may have also resulted from lower affinity of immune IgM to whole bacteria and/or diminished access of a bulky IgM molecule to the 2C7 epitope, which may be partially obscured by glycose extension that occurs beyond lactose substitution of HepI within the LOS core (Figure 1).

2C7 antibodies may shorten infection by enhancing complement activation on bacteria. Small amounts of complement [44] on mucosal surfaces may not be sufficient to kill gonococci directly but killing is amplified when complement receptors on professional phagocytes, mostly PMNs, are targeted in combination with Fc receptors to enhance uptake of opsonized gonococci [45], [46]. Although controversy remains about the fate of gonococci taken up by PMNs, gonococci that resist killing by PMNs are ingested principally via non-opsonic mechanisms [30]; gonococci that are taken up via opsonic mechanisms are readily killed [47].

Gonococci possess numerous phase-variable genes [48] that are responsible for antigenic diversity, a major obstacle for vaccine development. The 2C7 epitope requires expression of the phase-variable *lgtG* gene product (Figure 1). However, the majority of clinical isolates of *N. gonorrhoeae* express the 2C7 epitope [9] suggesting a critical role for this structure in gonococcal survival and pathogenesis. Our experiments showed durability/persistence of the 2C7 epitope despite selective immune pressure; FA1090 colonies isolated throughout the course of infection in immune mice always expressed the 2C7 epitope. Indeed, an *lgtG* deletion mutant of FA1090wt was more rapidly cleared in the murine infection model.

We acknowledge that mice immunized with MAP1 were not resistant to intravaginal challenge; rather they showed enhanced clearance of infection and diminished bacterial burdens. We note that fewer than 50% of immune animals cleared infection in the first 5 days and accelerated decreases in bacterial loads did not occur until after the third day of infection.

In conclusion, a vaccine that targets the 2C7 LOS epitope represents a significant step forward in the development of a vaccine against gonococcal infection. We are entering an era where conventional antibiotics against this infection have ceased to be universally effective. Public health measures, including immunization of high-risk individuals with a safe and effective vaccine to curb the spread of this disease are critical.

## Materials and Methods

### Ethics statement

Normal human serum samples were anonymized, pooled and used as a source of human complement in this study. Collection of sera and its use were approved by the University of Massachusetts Medical School Institutional Review Board (IRB) (Docket No. H-11733). Informed, written consent was obtained from all serum donors. Use of animals in this study was performed in strict accordance with the recommendations in the Guide for the Care and Use of Laboratory Animals of the National Institutes of Health. The protocol was approved by the Institutional Animal Care and Use Committee (IACUC) at the University of Massachusetts Medical School (Docket No. A-1931).

### Bacterial strains

*N. gonorrhoeae* FA1090wt (PorB1B, streptomycin resistant, serum resistant [SR]) [49], was used for mouse experimental challenge. To evaluate the contribution of the 2C7 LOS structure to gonococcal fitness in the mouse, an isogenic mutant that lacked the 2C7 epitope, FA1090*lgtG* ^−^, lacking HepII glycans and consequently the 2C7 epitope [12], was constructed and used for comparison. A 1.2 kb fragment of DNA containing *lgtG* was amplified from *N. gonorrhoeae* strain 398079 [50] using the primers lgtGF530 (CGCATTACCCTACCCCCTCACGCAC) and lgtGR1729 (TCTGTACGACGTTTTGAAAATTGC). The resultant amplicon was cloned into pCR2.1-TOPO (Invitrogen, Carlsbad, CA). The plasmid (pRYGW1) that was recovered was digested with *StuI* and ligated to a blunt 2.6 kb tetracycline resistance cassette containing *tetM* that had been excised from pHSX-tetM-lacIOP [51] using *HincII*. Linearized plasmid DNA was used to transform FA1090wt as described previously [52]. Transformants were selected on GC agar base media (Remel, Lenexa, KS) containing Isovitalex equivalent and 0.2 µg/ml tetracycline. PCR was used to confirm the *lgtG::tet* genotype. FA1090wt and FA190*lgtG* ^−^ LOS structures were assessed by: silver staining of LOS separated by SDS-PAGE; western blot using mAbs 3F11, L1, L8 and 2C7 [18], [19], [20], [21]; compositional analysis by negative ion spectrometry and DNA sequencing of the homo-polymer repeats present in the phase-variable *lgtA*, *lgtC*, *lgtD* and *lgtG* genes.

### Monoclonal antibody (mAb)

mAb 2C7 (murine isotype IgG3λ) [9], directed against the 2C7 epitope was affinity purified as described previously [11].

### Human serum

Normal human sera (NHS) obtained from 13 healthy adult human volunteers were equally distributed into a pool, aliquoted and stored at −70°C.

### Multiple antigen peptide (MAP) synthesis

MAP1 (calculated molecular mass, 15,360 daltons) and MAP-control peptide (mass,16,176 daltons) were prepared and confirmed as described previously [11].

### Passive immunization of mice

Female BALB/c mice 5–6 wks of age (Jackson Laboratories) in the diestrus phase of the estrous cycle were started on treatment (that day) with 0.5 mg. of water soluble 17β-estradiol (Sigma) given subcutaneously on each of three days; −2, 0 and +2 days (before, the day of and after inoculation) to prolong the estrus phase of the cycle and promote susceptibility to *N. gonorrhoeae* infection. Antibiotics ineffective against *N. gonorrhoeae* were also used to reduce competitive microflora [53], [54] . Estradiol treated mice were given either mAb 2C7 or control IgG3λ mAb intraperitoneally (ip) at a dose of 20 µg (10 µg twice a day) for 3 consecutive days: 2 days prior to infection and the day of infection. Dosing of 2C7 mAb was determined *a priori* and yielded serum mAb levels of 1.04±0.06 µg/ml on the day of infection and the subsequent 4 days and vaginal levels of 0.01±0.004 µg/ml on the day of infection, the next day and 0.006±0.004 µg/ml on the third day (data not shown). IgG3λ mAb was administered using the same schedule.

### Active immunization of mice

Two groups of 32 female BALB/c mice (Jackson Laboratories), aged 5–6 weeks, were immunized intraperitoneally (ip) on the same day with either 50 µg of MAP1 or 50 µg of MAP-control, each emulsified in 75 µg of Monophosphoryl Lipid A (MPL)-containing adjuvant (Sigma Adjuvant System). Primary immunization was followed by 3 booster doses at 3 weekly intervals (Figure 2) and blood was collected 2 weeks following each boost for serology. These mice were used for studies of experimental infection.

### Fractionation of mouse immune serum

We passed aliquots (250 µl) of sera from 6 additional uninfected mice immunized with MAP1 over Protein A/G-agarose (Pierce). Flow through and eluent fractions were dialyzed (0.9% saline; Amicon 15 ultra device [30 kDa cutoff]) and concentrated back to original volumes. Anti-LOS IgG, IgM and IgA concentrations were determined by ELISA.

### Measurement of anti-LOS antibodies (mouse serum, vaginal washings and mAb 2C7) by ELISA

Mouse immune serum or mAb 2C7 each diluted in PBS containing 0.05% Tween 20 was dispensed into microtiter wells (Immulon 1B.) coated with whole bacterial lysates of FA1090wt (or mutant F1090*lgtG* ^−^) and ELISA performed [11]. Mouse vaginal cavities were swabbed with sterile polyester tipped applicators (Solon Manufacturing Company) that were rinsed/eluted in 100 µl normal saline and dispensed onto microtiter wells coated with whole bacterial (FA1090wt) lysates and ELISA performed [11].

### Measurement of LOS IgG subclass antibodies

LOS IgG subclass antibodies were quantified using a mouse isotype-specific ELISA kit (Bethyl Laboratories). Diluted immune serum was added to microtiter wells coated with whole bacterial lysates of FA1090wt and ELISA performed [11].

### Serum bactericidal assays

Serum bactericidal assays against FA1090wt [55] were performed with: i) mAb 2C7, ii) sera from 6 MAP1 immunized mice not challenged with *N. gonorrhoeae*, iii) immune serum IgG fractions eluted from protein A/G-agarose and iv) flow through fractions devoid of immune containing residual IgM + IgA. Pooled NHS was used as a source of complement.

### Intravaginal challenge

#### Non-immune mice

A dual/competitive infection experiment was performed using a mixture of FA1090wt and FA1090*lgtG* ^−^ to determine if the 2C7 LOS structure provided gonococci a survival advantage. Female BALB/c mice 5–6 wks of age (Jackson Laboratories) in the diestrus phase of the estrous cycle were treated with water soluble 17β-estradiol (Sigma) and antibiotics as described previously [53], [54] and the following day were vaginally inoculated with a mixture of FA1090wt (1.5×10^5^ CFU) and FA1090*lgtG* ^−^ (1.6×10^5^ CFU) on Day 0 and colony forming counts (CFUs) performed daily by duplicate plating. 100 µl vaginal swab rinses were quantitatively cultured for *N. gonorrhoeae* daily (43) onto GC agar supplemented with vancomycin, colistin, nystatin, trimethoprim and streptomycin (GC-VCNTS) and GC-VCNTS plus tetracycline (0.2 µg/ml); the latter permitted growth of FA1090 *lgtG* ^−^ but not the wt strain.

#### 2C7 mAb passively immunized animals

Female BALB/c mice 5–6 wks of age (Jackson Laboratories) in the diestrus phase of the estrous cycle were treated with water soluble 17β-estradiol (Sigma) and antibiotics as described above [53]. Mice were passively immunized with mAb 2C7 or IgG3λ mAb and were challenged intravaginally with FA1090wt (5.7×10^5^ CFU) on the following day (Day 0) and colony forming counts (CFUs) determined daily by duplicate plating of vaginal swab rinses as described above. A replicate experiment was performed on a second group a week later using the same number of animals and protocol used in the first group; animals were challenged with 3.6×10^5^ CFU of FA1090wt. Results in the two groups were analyzed separately. To assess durability of the 2C7 epitope under antibody pressure, colony blots were performed on all gonococcal colonies isolated from animals that remained infected in the replicate experiment on day 7 after inoculation. Colony containing agar plates were overlayed with nitrocellulose and transfer of colony material was allowed to proceed for at least 2 minutes or until the membranes were totally wet. Then the membranes were blotted gently with kimwipes 3 times using a fresh kimwipe each time. Membranes were then blocked with PBS-1% dry milk for 30 min at 24C and incubated with mAb 2C7 for 15 h at 4°C. Membrane-bound mAb 2C7 was incubated for one hour with alkaline phosphatase-conjugated anti-mouse IgG (Sigma) used at 1∶1000 dilution in PBS-1% dry milk followed by 5-bromo-4-chloro-3-indolyl phosphate/nitro blue tetrazolium (BCIP/NBT) for detection.

To test mAb 2C7 specificity, passively immunized mice inoculated with mutant FA1090*lgtG* ^−^ were also examined.

#### MAP1 actively immunized animals

At fourteen weeks after immunization, we identified immunized mice that had entered the diestrus phase the day before challenge was to be performed; diestrus is asynchronous and not all of the animals entered the diestrus phase at the same time. Immune mice that were in the diestrus phase of the cycle were treated with water soluble 17β-estradiol (Sigma) as above to prolong the estrous phase of the cycle and promote susceptibility to *N. gonorrhoeae*. Antibiotics were also used to reduce competitive microflora [53], [54]. Half [16 animals] of each group that had been immunized with MAP1 or MAP-control, were challenged at this time (Group 1 in Figure 5) and colony forming counts (CFUs) determined daily by duplicate plating of vaginal swab rinses as described above. A week later, using the identical protocol that identified and treated Group 1 animals, we challenged as many of the remaining immunized mice; 10 immunized with MAP1 and 7 with MAP-control, that had entered the diestrus phase the day before challenge (Group 2 in figure 5). Results in the two groups were analyzed separately. By performing challenge experiments at two separate times, we used more animals, thereby gaining maximum usage of the immunized animals.

Group 1 animals consisted of estrogen treated immune mice (MAP1 (n = 16) and MAP-control (n = 16)) that were challenged intravaginally with FA1090wt (5.4×10^5^ CFU) that expresses the 2C7 epitope (Figure 5). Group 2 animals, MAP1 (n = 10) and MAP-control (n = 7)) immunized mice were similarly treated with estrogen and were challenged intravaginally with FA1090wt (4×10^5^ CFU) (Figure 2). In all experiments, vaginal mucus was quantitatively cultured on GC-VCNTS daily for *N. gonorrhoeae* as described above [53].

### Statistical analysis

Experiments that compared time to clearance of *N. gonorrhoeae* CFU in two independent groups of mice estimated and tested three characteristics of the data: Time to clearance, longitudinal trends in mean log_10_ CFU and the cumulative CFU as area under the curve (AUC). Statistical analyses were performed using mice that initially yielded bacterial colonies on Days 1 and/or 2. Median time to clearance was estimated using Kaplan-Meier survival curves; the times to clearance were compared between groups using a log-rank test. Mean log_10_ CFU trends over time were compared between groups using a linear mixed model with mouse as the random effect using both a random intercept and a random slope. A quadratic function in time was determined to provide the best fit; random slopes were also quadratic in time. A likelihood ratio test was used to compare nested models (with and without the interaction term of group and time) to test whether the trend differed over time between the two groups. The mean area under the curve (log_10_CFU vs. time) was computed for each mouse to estimate the bacterial burden over time (cumulative infection); the means under the curves were compared between groups using the nonparametric rank sum test because distributions were skewed or kurtotic.

In the experiment that used unimmunized animals challenged with strains FA1090 wt and FA1090 *lgtG* ^−^ simultaneously and in passive and active immunization experiments that used strains individually the data were paired. Individual Kaplan-Meier curves were estimated with median time to clearance. Paired times to clearance were tested using a signed rank to test the paired differences. Mean log_10_ CFU trends over time were compared by taking the difference between wt and mutant within each mouse at each day and modeling the difference over time, testing if the difference was significantly different than zero using a mixed model with mouse as a random effect. The mean area under the curve for wt and mutant were computed separately and paired differences were compared using a signed rank test.

Measurement and comparison of serum IgG anti-LOS levels at weeks 5, 8, 11 and 14 after immunization with MAP1 were carried out using linear mixed models with mouse as the random effect (random intercept and slope). A Wald chi-square tested the overall significance of antibody levels at each of the weeks when measurements were made; estimates of differences in levels between adjacent times (weeks) used the coefficients of the model.

The association of burden of infection as measured by AUC of log_10_CFU vs. time with vaginal antibody concentration in the combined experiments was tested using linear regression analysis stratified by experiment.

## Supporting Information

Figure S1 **Selective survival of FA1090wt (1.5×10^5^ CFU) and FA1090** ***lgtG*** **^−^ (1.6×10^5^ CFU) mixed in equal proportions and inoculated into mice.** ***A*** **.** Kaplan Meier analysis of time to clearance showing differences in clearance of FA1090wt (red solid line) and FA1090*lgtG* ^−^ (blue dotted line) mixed together; ***B*** **.** Colonization (Log_10_ CFU) at daily intervals of FA1090wt and FA1090*lgtG* ^−^ , limit of detection, <5 CFUs; ***C.*** Bacterial burdens consolidated over time (Area Under the Curve analysis) of FA1090wt and FA1090*lgtG* ^−^.
(TIF)

Figure S2 **Survival of FA1090** ***lgtG*** **^−^ inoculated into mice passively immunized with mAb 2C7 (dashed line) vs. control immunization (solid line).** ***A.*** Kaplan Meier analysis of time to clearance showing differences in clearance of mAb 2C7 (dashed line) vs. control IgG3λ mAb (solid line) treated animals; ***B.*** Colonization (Log_10_ CFU) at daily intervals of mAb 2C7 (dashed line) vs. control IgG3λ mAb (solid line) treated animals; ***C.*** Bacterial burdens consolidated over time (Area Under the Curve analysis) of mAb 2C7 vs. control IgG3λ mAb treated animals.
(TIF)

Figure S3 **Anti-IgG LOS antibody responses induced by MAP1-MPL immunization.** 32 BALB/c mice were immunized intraperitoneally (ip) with MAP1 emulsified with MPL and boosted four times at 3-week intervals. Total anti-LOS IgG antibody levels at wks 5, 8, 11 and 14 following primary immunization are shown. Mixed model analysis of mean anti-LOS antibody levels over time showed significant increases between weeks designated for antibody testing, comparing antibody levels on week 5 vs. 8, week 8 vs.11 and week 11 vs. 14 (p<0.001).
(TIF)

Table S1 **Comparison of serum immunoglobulin isotype-specific anti-LOS concentrations of immunized mice used for challenge versus identically immunized mice used only for anti-LOS measurements and bactericidal assays**.
(DOC)

Table S2 **Mean Differences (and 95% CI) of serum immunoglobulin isotype-specific anti-LOS concentrations between immunized mice used for challenge versus identically immunized mice used only for anti-LOS measurements and bactericidal assays.**
(DOC)

Table S3 **Anti-LOS IgG, IgM and IgA concentrations in sera of 6 mice immunized with MAP1-MPL and their protein A/G fractionates.**
(DOC)
