# Supplementary figures and images for: Immunization against a Saccharide Epitope Accelerates Clearance of Experimental Gonococcal Infection
Source: PLoS Pathog. 2013 Aug 29;9(8):e1003559. doi: 10.1371/journal.ppat.1003559 (PMC3757034; doi:10.1371/journal.ppat.1003559)

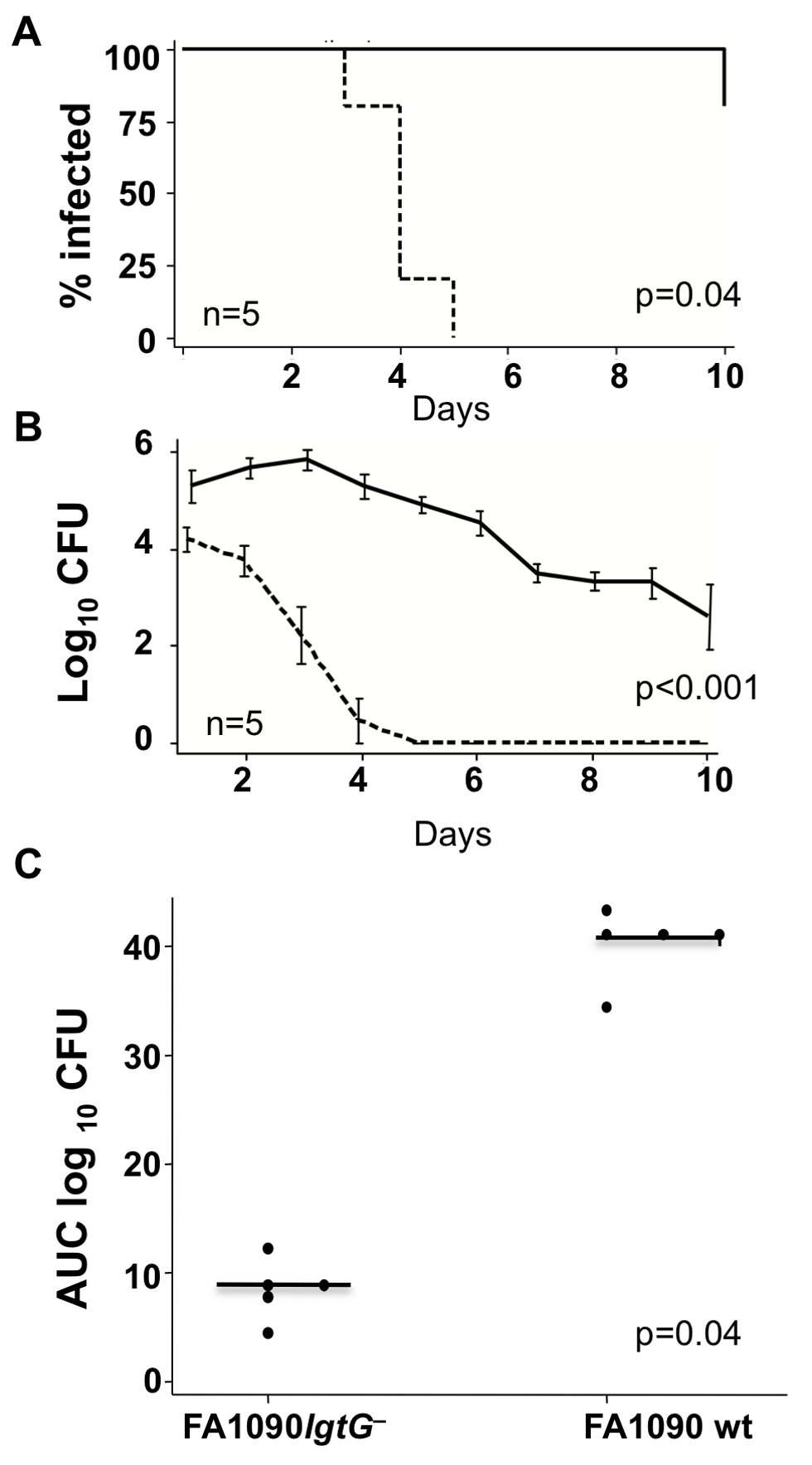

Supplement: Figure S1 — Selective survival of FA1090wt (1.5×10^5^ CFU) and FA1090 lgtG − (1.6×10^5^ CFU) mixed in equal proportions and inoculated into mice. A . Kaplan Meier analysis of time to clearance showing differences in clearance of FA1090wt (red solid line) and FA1090lgtG − (blue dotted line) mixed together; B . Colonization (Log10 CFU) at daily intervals of FA1090wt and FA1090lgtG − , limit of detection, <5 CFUs; C. Bacterial burdens consolidated over time (Area Under the Curve analysis) of FA1090wt and FA1090lgtG −. (TIF) [file ppat.1003559.s001.tif]

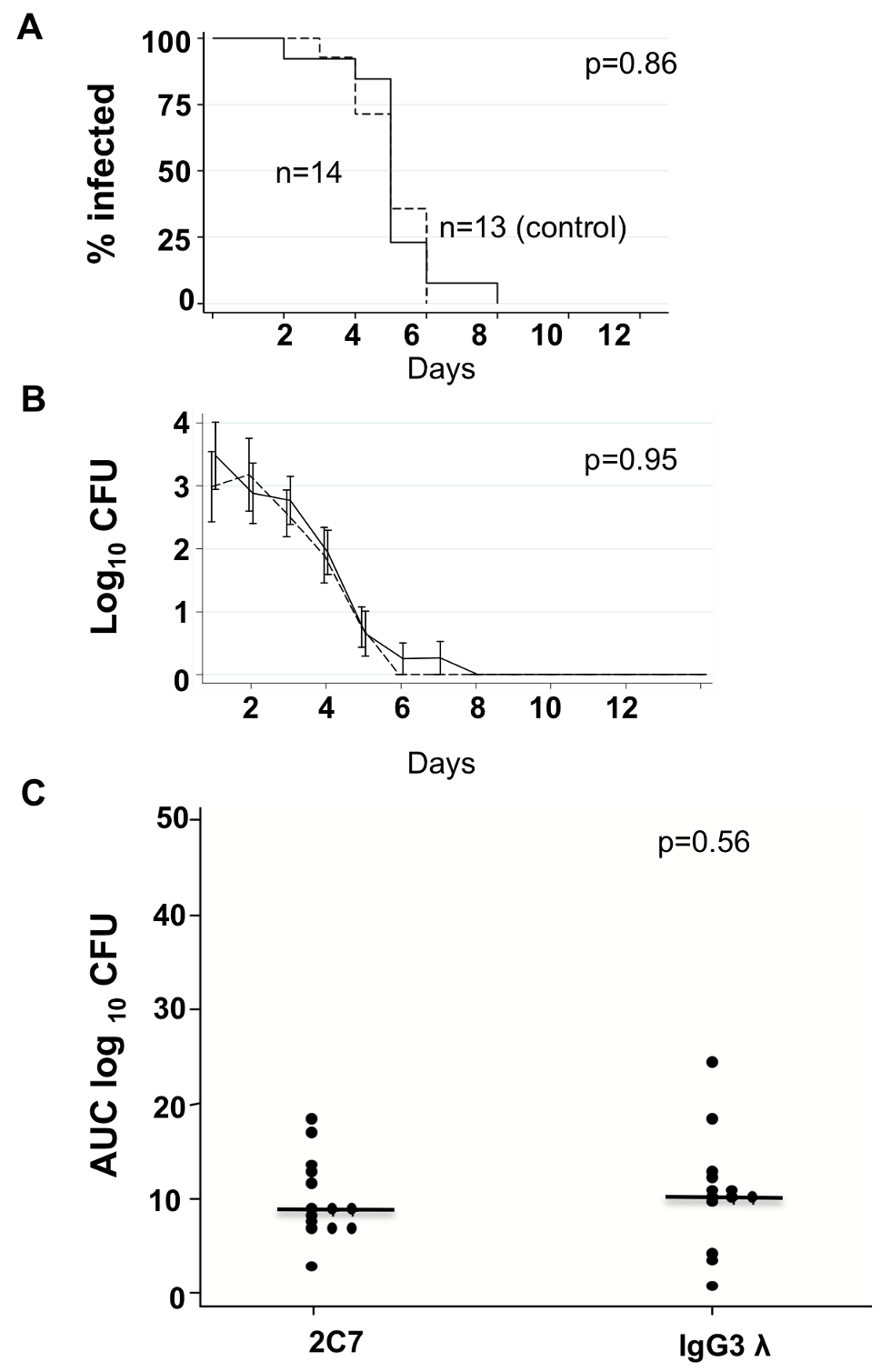

Supplement: Figure S2 — Survival of FA1090 lgtG − inoculated into mice passively immunized with mAb 2C7 (dashed line) vs. control immunization (solid line). A. Kaplan Meier analysis of time to clearance showing differences in clearance of mAb 2C7 (dashed line) vs. control IgG3λ mAb (solid line) treated animals; B. Colonization (Log10 CFU) at daily intervals of mAb 2C7 (dashed line) vs. control IgG3λ mAb (solid line) treated animals; C. Bacterial burdens consolidated over time (Area Under the Curve analysis) of mAb 2C7 vs. control IgG3λ mAb treated animals. (TIF) [file ppat.1003559.s002.tif]

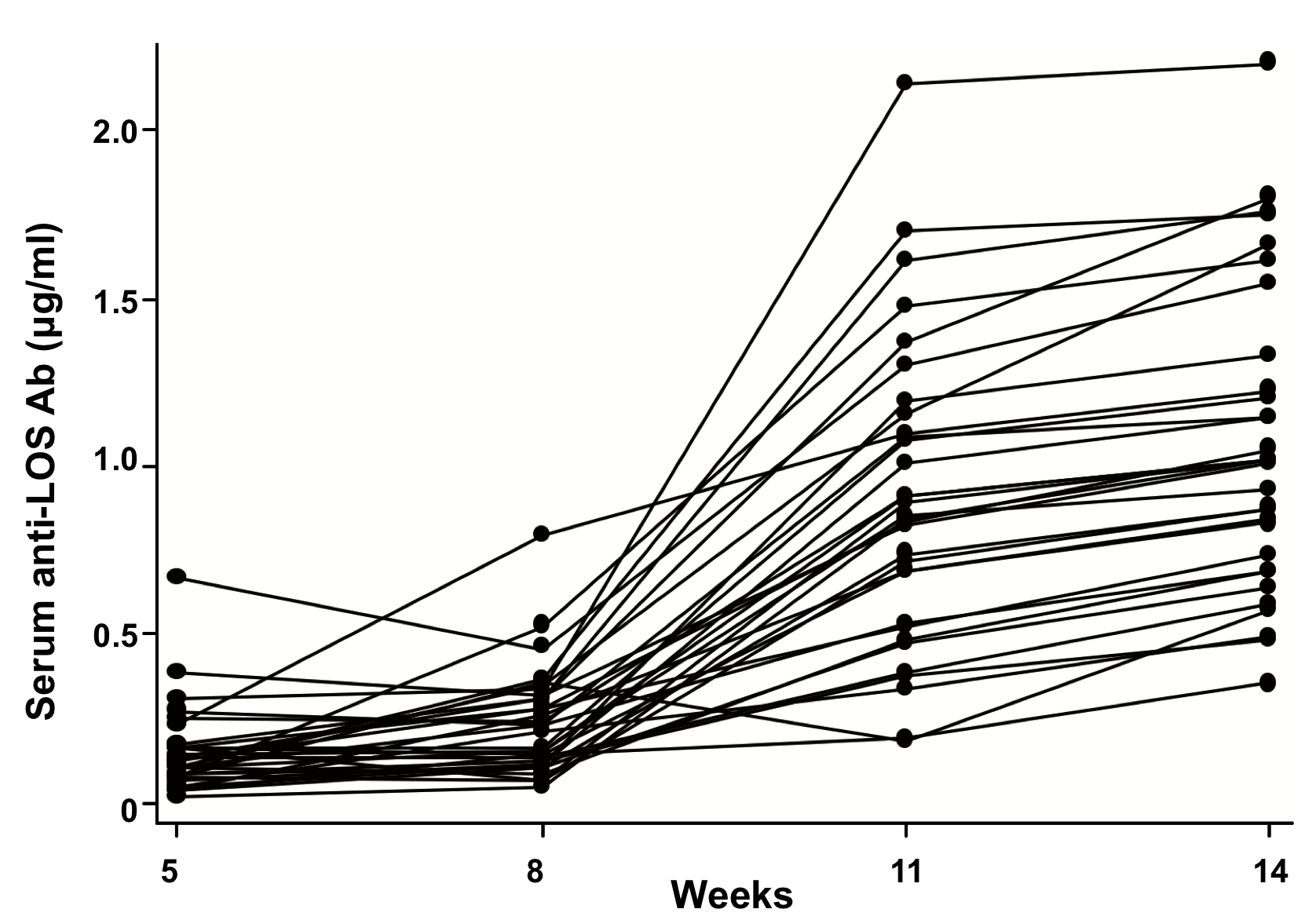

Supplement: Figure S3 — Anti-IgG LOS antibody responses induced by MAP1-MPL immunization. 32 BALB/c mice were immunized intraperitoneally (ip) with MAP1 emulsified with MPL and boosted four times at 3-week intervals. Total anti-LOS IgG antibody levels at wks 5, 8, 11 and 14 following primary immunization are shown. Mixed model analysis of mean anti-LOS antibody levels over time showed significant increases between weeks designated for antibody testing, comparing antibody levels on week 5 vs. 8, week 8 vs.11 and week 11 vs. 14 (p<0.001). (TIF) [file ppat.1003559.s003.tif]
