# Supplementary material for: Immunization against a Saccharide Epitope Accelerates Clearance of Experimental Gonococcal Infection
Source: PLoS Pathog. 2013 Aug 29;9(8):e1003559. doi: 10.1371/journal.ppat.1003559 (PMC3757034; doi:10.1371/journal.ppat.1003559)
Supplement: Table S1 — Comparison of serum immunoglobulin isotype-specific anti-LOS concentrations of immunized mice used for challenge versus identically immunized mice used only for anti-LOS measurements and bactericidal assays. (DOC) [file ppat.1003559.s004.doc]

**Table S1**

|  | **Used for Challenge** | | | | | **Used for Bactericidal Assay** | | | | |  |
| --- | --- | --- | --- | --- | --- | --- | --- | --- | --- | --- | --- |
|  | n=26 | | | | | n=6 | | | | |  |
| Variable | Mean | Std. Dev. | Median | IQR | | Mean | Std. Dev. | Median | IQR | | p* |
| IgG | 1.06 | 0.46 | 1.02 | 0.72 | 1.38 | 1.08 | 0.42 | 1.08 | 0.67 | 1.35 | 0.81 |
| IgG1 | 0.28 | 0.13 | 0.26 | 0.17 | 0.36 | 0.26 | 0.05 | 0.25 | 0.21 | 0.31 | 0.83 |
| IgG2a | 0.55 | 0.23 | 0.52 | 0.35 | 0.71 | 0.46 | 0.23 | 0.39 | 0.32 | 0.58 | 0.36 |
| IgG2b | 0.38 | 0.23 | 0.35 | 0.20 | 0.45 | 0.33 | 0.24 | 0.27 | 0.14 | 0.50 | 0.50 |
| IgG3 | 0.53 | 0.28 | 0.45 | 0.28 | 0.72 | 0.36 | 0.20 | 0.29 | 0.20 | 0.51 | 0.12 |
| IgM | 4.02 | 1.87 | 3.58 | 2.70 | 5.41 | 3.03 | 2.28 | 2.77 | 1.38 | 4.47 | 0.33 |
| IgA | 0.35 | 0.16 | 0.32 | 0.22 | 0.47 | 0.29 | 0.15 | 0.24 | 0.20 | 0.38 | 0.48 |

*p value from the Kruskall-Wallis rank sum test
