# Supplementary material for: Immunization against a Saccharide Epitope Accelerates Clearance of Experimental Gonococcal Infection
Source: PLoS Pathog. 2013 Aug 29;9(8):e1003559. doi: 10.1371/journal.ppat.1003559 (PMC3757034; doi:10.1371/journal.ppat.1003559)
Supplement: Table S2 — Mean Differences (and 95% CI) of serum immunoglobulin isotype-specific anti-LOS concentrations between immunized mice used for challenge versus identically immunized mice used only for anti-LOS measurements and bactericidal assays. (DOC) [file ppat.1003559.s005.doc]

**Table S2**

| Variable | Mean Difference (M_26_-M_6_) | SE | 95% CI  lb ub | |
| --- | --- | --- | --- | --- |
| IgG | -0.02 | 0.21 | -0.44 | 0.40 |
| IgG1 | 0.02 | 0.06 | -0.09 | 0.14 |
| IgG2a | 0.09 | 0.10 | -0.12 | 0.30 |
| IgG2b | 0.04 | 0.11 | -0.17 | 0.26 |
| IgG3 | 0.17 | 0.12 | -0.08 | 0.42 |
| IgM | 0.99 | 0.88 | -0.81 | 2.79 |
| IgA | 0.06 | 0.07 | -0.09 | 0.20 |
