# Supplementary material for: Immunization against a Saccharide Epitope Accelerates Clearance of Experimental Gonococcal Infection
Source: PLoS Pathog. 2013 Aug 29;9(8):e1003559. doi: 10.1371/journal.ppat.1003559 (PMC3757034; doi:10.1371/journal.ppat.1003559)
Supplement: Table S3 — Anti-LOS IgG, IgM and IgA concentrations in sera of 6 mice immunized with MAP1-MPL and their protein A/G fractionates. (DOC) [file ppat.1003559.s006.doc]

**Table S3**

|  | **Mouse #** | | | | | | | | | | | | | | | | | |
| --- | --- | --- | --- | --- | --- | --- | --- | --- | --- | --- | --- | --- | --- | --- | --- | --- | --- | --- |
|  | **1** | | | **2** | | | **3** | | | **4** | | | **5** | | | **6** | | |
|  | **Anti-LOS concentration (µg/ml)** | | | | | | | | | | | | | | | | | |
|  | **IgG** | **IgM** | **IgA** | **IgG** | **IgM** | **IgA** | **IgG** | **IgM** | **IgA** | **IgG** | **IgM** | **IgA** | **IgG** | **IgM** | **IgA** | **IgG** | **IgM** | **IgA** |
| **Serum** | 1.593 | 6.944 | 0.583 | 1.163 | 1.732 | 0.222 | 1.174 | 0.332 | 0.317 | 1.086 | 3.648 | 0.244 | 1.111 | 3.552 | 0.244 | 0.811 | 1.998 | 0.147 |
| **Flow-through** | 0.000 | 6.673 | 0.504 | 0.000 | 1.681 | 0.200 | 0.000 | 0.259 | 0.248 | 0.000 | 3.440 | 0.189 | 0.000 | 3.755 | 0.200 | 0.000 | 2.151 | 0.104 |
| **Elute** | 1.453 | 0.833 | 0.033 | 1.156 | 0.166 | 0.007 | 1.185 | 0.159 | 0.070 | 1.130 | 0.429 | 0.004 | 1.100 | 0.351 | 0.033 | 0.761 | 0.288 | 0.022 |
